# Why Does Public Transport Not Arrive on Time? The Pervasiveness of Equal Headway Instability

**DOI:** 10.1371/journal.pone.0007292

**Published:** 2009-10-28

**Authors:** Carlos Gershenson, Luis A. Pineda

**Affiliations:** Computer Sciences Department, Instituto de Investigaciones en Matemáticas Aplicadas y en Sistemas, Universidad Nacional Autónoma de México, Ciudad Universitaria, México D.F., México; University of East Piedmont, Italy

## Abstract

**Background:**

The equal headway instability phenomenon is pervasive in public transport systems. This instability is characterized by an aggregation of vehicles that causes inefficient service. While equal headway instability is common, it has not been studied independently of a particular scenario. However, the phenomenon is apparent in many transport systems and can be modeled and rectified in abstraction.

**Methodology:**

We present a multi-agent simulation where a *default* method with no restrictions always leads to unstable headways. We discuss two methods that attempt to achieve equal headways, called *minimum* and *maximum*. Since one parameter of the methods depends on the passenger density, adaptive versions—where the relevant parameter is adjusted automatically—are also put forward. Our results show that the *adaptive maximum* method improves significantly over the *default* method. The model and simulation give insights of the interplay between transport design and passenger behavior. Finally, we provide technological and social suggestions for engineers and passengers to help achieve equal headways and thus reduce delays.

**Conclusions:**

The equal headway instability phenomenon can be avoided with the suggested technological and social measures.

## Introduction

**Figure 1:**
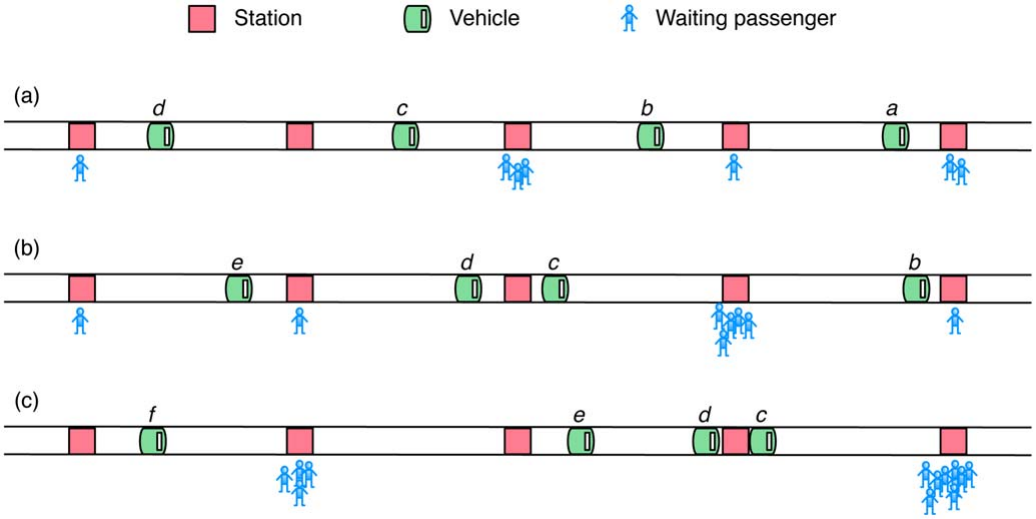
Illustration of equal headway instability. a) Vehicles with a homogeneous temporal distribution, *i.e.* equal headways. Passengers arriving at random cause some stations to have more demand than others. b) Vehicle 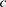 is delayed after serving a busy station. This causes a longer waiting time at the next station, leading to a higher demand ahead of 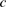. Also, vehicle 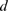 faces less demand, approaching 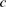. c) Vehicle 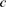 is delayed even more and vehicles 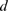 and 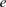 aggregate behind it, forming a platoon. There is a separation between 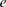 and 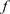, making it likely that 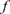 will encounter busy stations ahead of it. This configuration causes longer waiting times for passengers at stations, higher demands at each stop, and increased vehicle travel times. The average service frequency at stations is much slower for platoons than for vehicles with an equal headway.

It is well known that public transport passengers arriving randomly at stations are served best when the time intervals between vehicles—also known as the headway—are equal [1, p. 133]. In other words, the passing of vehicles at stations is regular. This minimizes waiting times for passengers at stations. However, the configuration where the headways are equal is unstable. This is because of the following: if one vehicle is delayed, then there will be a shorter headway with the vehicle behind and a longer headway with the vehicle in front. Longer headways lead to more passengers waiting at stations, which lead to more delays. Also, shorter headways lead to less passengers waiting. Thus, vehicles moving behind a delayed vehicle will go faster than average. Even if a minimum waiting time at stations is established, during times of high passenger demand, slower vehicles will be reached by faster ones. After some time, several vehicles will be “platooning”, *i.e.* traveling together. This makes the service inefficient, since people need to wait more time for a platoon to arrive than if the vehicles were equally spaced in time. Moreover, when a platoon arrives at a station, there will be much more people waiting, delaying the platoon flow. Figure 1 illustrates this phenomenon.

We can distinguish two causes of losing equal headways:

1. *Vehicles go faster than expected*. In principle, this can easily be rectified by forcing vehicles to wait at stations until their expected departure time comes. In practice, some conductors might be reluctant to abide this restriction.
2. *Vehicles go slower than expected*. This is more complicated to solve, since in most cases the causes of the delay are external to the vehicles. Depending on the type of transport, these can be heavy traffic, poorly synchronized traffic lights, and passenger behaviors.

The problem of having an equal headway instability is that it makes transport inefficient. Many vehicles are used below their capacity and adding more vehicles does not improve the situation, as they simply aggregate to platoons. This leads to large wastes of infrastructure and fuel. Moreover, from the passenger's viewpoint, platoons of public transport cause greater delays and make travel less comfortable, as many passengers accumulate within few vehicles.

Among the transportation systems that present equal headway instability, we can distinguish two types: those that allow passing and those that do not. In the latter category (no passing), there are metros, trams, some trains, and bus rapid transit [2]. In the former category (passing allowed), there are buses [3], some trains, and elevators [4], [5]. Even when passing is allowed, equal headway instability is observed: If a fast vehicle passes a slow one, it will encounter at the next stop a higher passenger volume, delaying its route. Therefore, another vehicle will pass again the vehicle in front. There will be a shuffling of positions within the platoon, but grouping is a stable configuration, while equal headways is an unstable one.

Since the equal headway instability is a general phenomenon, there have been several strategies proposed over the years in particular domains, such as metros [6], buses [7], [8], and elevators [9]. For example, sometimes at rush hour in the Red Line of the Boston metro (also known as “the T”, operated by the Massachusetts Bay Transport Authority), a delayed northbound train will announce at Park St. station that it will not stop until Harvard Sq. station. Thus, people who need a station before Harvard Sq. exit the train at Park St. station and wait for the next train, which comes close behind with free space. Like this, the delayed train can transfer some of its load to the train behind it, trying to regain equal headways. Independently of the inconveniences of this approach, it would be desirable to prevent the equal headway instability altogether, instead of trying to restore equal headways once they have been broken.

In this paper, we focus on equal headway instability as a phenomenon, studying it with a simple computational model, presented in the following section. Next, we show simulation results of an implementation in a multi-agent simulation: a *default* method always exhibits equal headway instability. We study two methods that restrict vehicle behavior to attempt to achieve equal headways. An adaptive version of these methods is also put forward. Theoretically, one of the adaptive methods always achieves equal headway stability unless the passenger density saturates the system. However, our discussion indicates that equal headway instability does not depend only on the method regulating the public transport, but also depends on the passenger behaviors. Recommendations for passengers and engineers follows. An important thing to notice is that equal headway instability can be avoided with appropriate passenger behaviors, not only technological sophistications. Concluding remarks and delineations for future work close the paper.

## Methods

Recently there has been an increased interest in modeling transport systems [10]–[15]. We developed a model to study the main characteristics of equal headway instability in public transport systems.

The simplest case is a metro-style system and we will restrict ourselves to it for the rest of the paper. Since speed and acceleration is regulated, and sometimes automatized, if everything works normally, only time spent at stations can cause delay [1]. This time will depend basically on passengers: how many are exiting, how many are entering, and how efficiently they are doing so. Buses (including rapid transit) are also delayed by traffic lights. Buses sharing streets with other vehicles are moreover affected by common traffic.

### Model

Our model uses abstract discrete time and space. Time 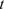 is measured in “ticks” and space is measured in vehicle lengths 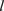, *i.e.* an abstract measure representing the length of one vehicle. In the model, there is a single cyclic lane of traffic, with a set 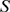 of stations and a set 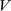 of vehicles servicing passengers that board at one station and leave at another station chosen randomly. The inflow of passengers is random, where the intervals between the arrivals of passengers at each station have a Poisson distribution with a mean of 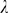 ticks. For example, if 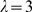, on average a new passenger will arrive at each station every three ticks, *i.e.* 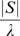 new passengers per tick for the whole system on average.

A vehicle 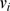 flows at a cruise velocity unless it reaches a station 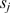 or another vehicle is in front closer or equal than a minimum separation distance 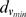. Removing this last restriction models transport systems that allow passing. At a station, passengers onboard the vehicle 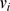 scheduled to exit at station 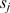 leave the vehicle with a random order, taking one tick each. Once all scheduled passengers leave the vehicle, passengers waiting at the station board the vehicle with a random order, also taking one tick each. The vehicle leaves the station when there are no more passengers waiting to board or the maximum passenger capacity 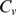 of the vehicle is reached. We assume stations have an infinite capacity 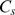, *i.e.* there can always be more passengers arriving.

### Validation

We do not need to model several wagons per vehicle, nor even several doors, since these are just scalings of the basic case of one door per vehicle.

Stations can be spaced homogeneously or not. In the former case, there is an equal interstation distance 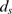 for all stations. Equal headway occurs when the intervehicle times 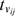 are equal. These can be plotted in histograms, but a more concise measure of the equal headway can be obtained with the standard deviation of the distribution of 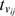, which is equivalent to the distribution of intervehicle frequencies 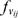 at a station chosen randomly. If this standard deviation 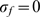, then all vehicles have an equal headway, *i.e.* 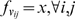. We can also study the standard deviation of the vehicle capacity usage 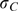. This reflects how evenly the passengers are distributed among the vehicles. If 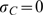, then all vehicles have the same capacity usage. As 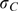 increases, it implies that some vehicles are more full and some are more empty. This unbalance is a property of the equal headway instability.

The performance of the system can be measured in several ways. We can look at the average travel times of vehicles or the average travel times of passengers. Since the dynamics are simplified, it is more illustrative to look at delays. This can be measured as the actual travel time minus the minimum possible travel time. Delays can be calculated for vehicles or passengers. Vehicle delay 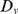 increases when stopped at stations, or between stations, waiting for another vehicle to move. 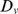 values are reset each time a vehicle goes around the cyclic track. Passenger delay 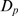 increases while waiting at departure stations (for arrival and boarding), within vehicles that are being delayed (see above), or while waiting to exit a vehicle at their destination station.

At its simplest case, the main dynamics of the model could be implemented with an elementary cellular automaton [16], [17], specifically rule 184 [18]–[20] with further restrictions for delays at stations. However, a multi-agent description is more explicit and is easier to extend, *e.g.* to allow passing. We used the NetLogo environment [21] to implement the model. The reader is invited to explore this simulation via web browser at the URL http://turing.iimas.unam.mx/~cgg/NetLogo/metro.html (or for short, http://tinyurl.com/EqHeIn).

**Figure 2:**
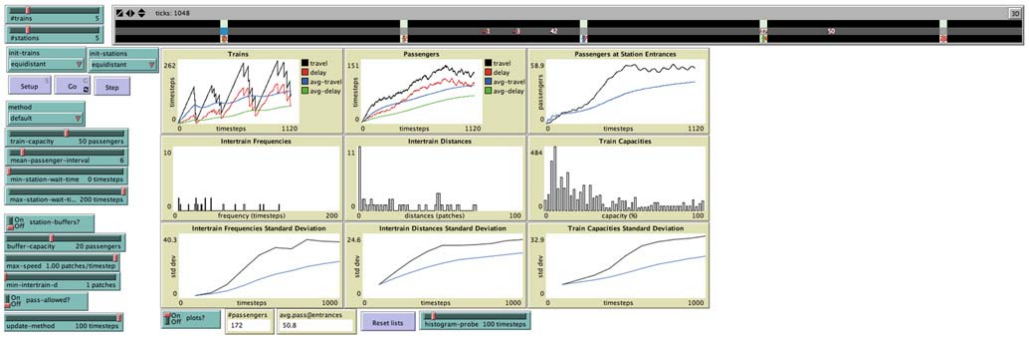
Screenshot of the simulation with equal headway instability, 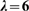. The track, vehicles and passengers are shown on the top, where numbers indicate the passengers in vehicles or waiting at stations. Different parameters of the simulation (some not relevant to the results presented here) can be adjusted. Different monitors, plots, and histograms show results in real time. The equal headway instability can be seen most clearly in the histograms: the intervehicle frequencies are irregular, as well as the intervehicle distances (with a sharp peak at 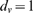, *i.e.* vehicles aggregated next to each other). This irregularity leads to high standard deviations. Notice also that the vehicle utilization (percentage of vehicle capacity used) has a very broad distribution, *i.e.* there are some vehicles working at almost full capacity, while many vehicles have a very low capacity utilization.

**Figure 3:**
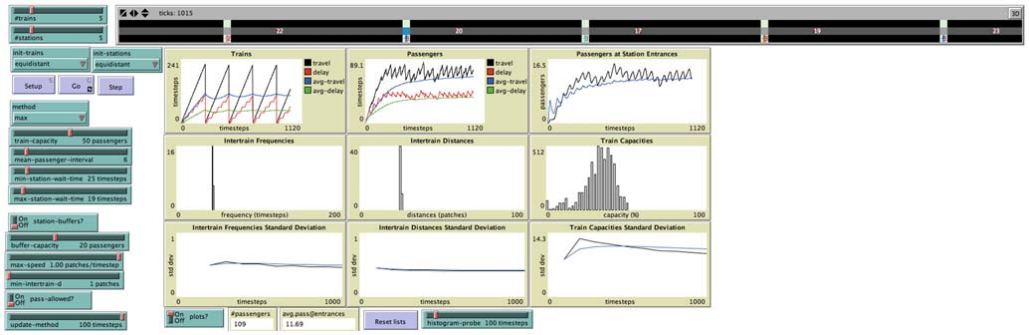
Screenshot of the simulation with equal headway stability, 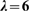. Notice that the intervehicle frequencies and distances are regular, shown with a peak in the histograms and low standard deviations (notice 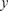-axis scale). Because of this regularity, the vehicle utilization histogram has a bell-shaped distribution, which reflects the passenger inflow interval probabilities.

A screenshot of the simulation where the vehicles reach an equal headway instability configuration is shown in Figure 2. A screenshot where equal headways are maintained (using an adaptive method described below) is shown in Figure 3.

Table 1 lists the model variables and the values used in the simulations.

**Table 1:** Model parameters and values used in simulation.

Variable	Description	Value
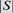	number of stations	5
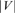	number of vehicles	5, [2], [8]
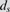	interstation distance (homogeneous)	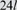
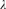	mean passenger inflow interval	[3], [15]
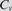	vehicle capacity	50 passengers
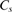	station capacity	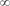
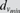	minimum separation distance	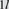
	vehicle cruise velocity	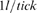
	vehicle acceleration	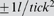

As described above, the model represents a metro-style system. It can be modified to allow passings and extended to include traffic lights and other traffic.

## Results

We used a scenario with five stations spaced homogeneously in a cyclic track of 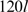. All stimulations started with empty vehicles and stations (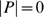). The vehicles start positioned between stations with an equal headway. Simulations ran for ten thousand ticks, unless a maximum number of passengers 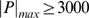 was reached.

For each value of 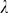, fifty simulations were run and aggregated in boxplotsA boxplot is a non-parametric representation of a statistical distribution. Each box contains the following information: The median (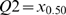) is represented by the horizontal line inside the box. The lower edge of the box represents the lower quartile (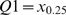) and the upper edge represents the upper quartile (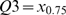). The interquartile range (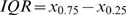) is represented by the height of the box. Data which is lesser than 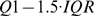 or greater than 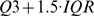 is considered an “outlier”, and is indicated with circles. The “whiskers” (horizontal lines connected to the box) show the smallest and largest values that are not outliers. Some of the boxplots presented in this paper have notches, which extend to 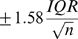, where 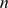 is the number of samples per box.

The results shown—except for the final number of passengers—are averaged over all of the simulation run, transients included. This was because for some cases, the equal headway is maintained for some time and then it collapses. We were interested in how fast this collapse occurs, which reflects the stability of the method and this requires averaging from the initial state of each run. Moreover, after the maximum passenger capacity of the system is exceeded, the system will not settle in a characteristic configuration that balances the passenger demand and the system capacity, as more and more passengers accumulate. Thus, it is not possible to decide at which point to exclude transient data, since for these cases all data is transient.

For each set of simulations, we present the mean passenger delay 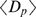, the mean vehicle delay 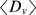, the mean standard deviation of intervehicle frequencies 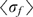, the final number of passengers 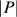, the mean standard deviation of vehicle capacity usage 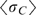, and the mean number of passengers at stations 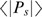. We consider unstable headways when 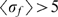 and a poor system performance when 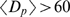 ticks.

### The *default* method

**Figure 4:**
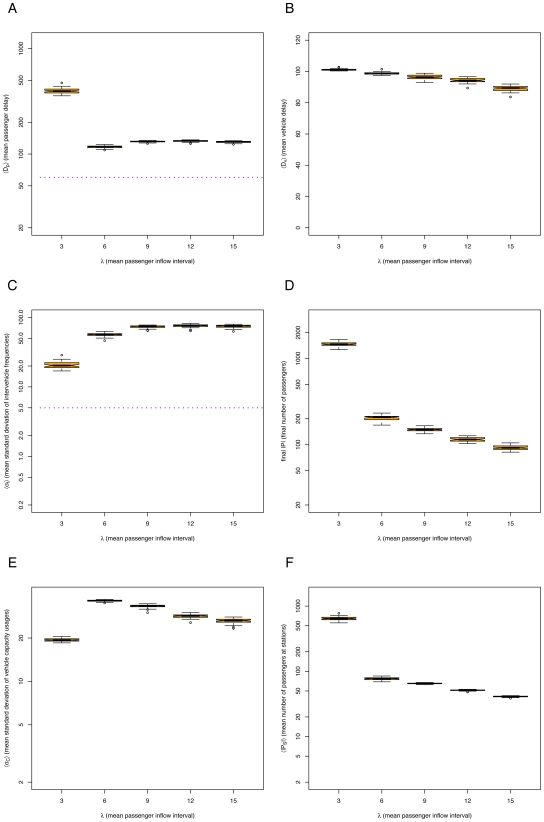
Simulation results for *default* method, varying mean passenger inflow intervals 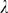 and number of vehicles 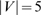: (A) mean passenger delay 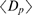 (data above horizontal dotted line indicates poor system performance), (B) mean vehicle delay 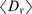, (C) mean standard deviation of intervehicle frequencies 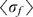 (data above horizontal dotted line implies unstable headways), (D) final number of passengers 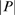, (E) mean standard deviation of vehicle capacity usage 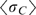, and (F) mean number of passengers at stations 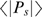. Notice log scale on 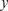 axis of all plots but (B).

We explored different restrictions to try to regulate an equal headway. The *default* case is without restrictions. In this case, the equal headway is always unstable (See Figure 4, in particular, the high standard deviations of intervehicle frequencies 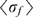 shown in Figure 4C). Since there are no restrictions on the vehicles, some will spend less time at stations than others, varying their headways. This causes the aggregation of vehicles, where the first one has a heavy passenger load and the following ones have a light passenger load, as indicated by the high standard deviation of vehicle capacity usage 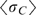 (Figure 4E). Thus, all the vehicles go at the speed of the slowest vehicle. Even if the passenger density is not so high (*e.g.* 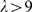), the performance of the system will be comparable to that of a high density (below saturation, 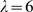, See Figures 4A and 4B), as the slowest vehicle will suffer from an unbalanced load. Notice that for 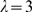 the system saturates, i.e. it is unable to serve the amount of incoming passengers, indicated by the high number of passengers in Figures 4D and 4F. For the *default* method, when there is a very high passenger demand, the headways are actually less unstable than with a low passenger demand (See Figure 4C). This is because vehicles reach their full capacity before all passengers at a station can be served. Thus, empty vehicles behind them separate from the full vehicles while waiting passengers to board. In this way, empty vehicles share some of the system's load, making the vehicle capacity usages slightly less unbalanced (See Figure 4E).

### The *minimum* method

To improve the system performance, one option is to add a *minimum* station waiting time 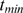, that will ensure that a vehicle will not leave the station even if there are no passengers waiting. This attacks the problem of vehicles going faster than expected. We call this restriction the *minimum* method, similar to the holding strategies described in [22]. The aim of this is to restrict faster vehicles from reaching slower ones.

**Figure 5:**
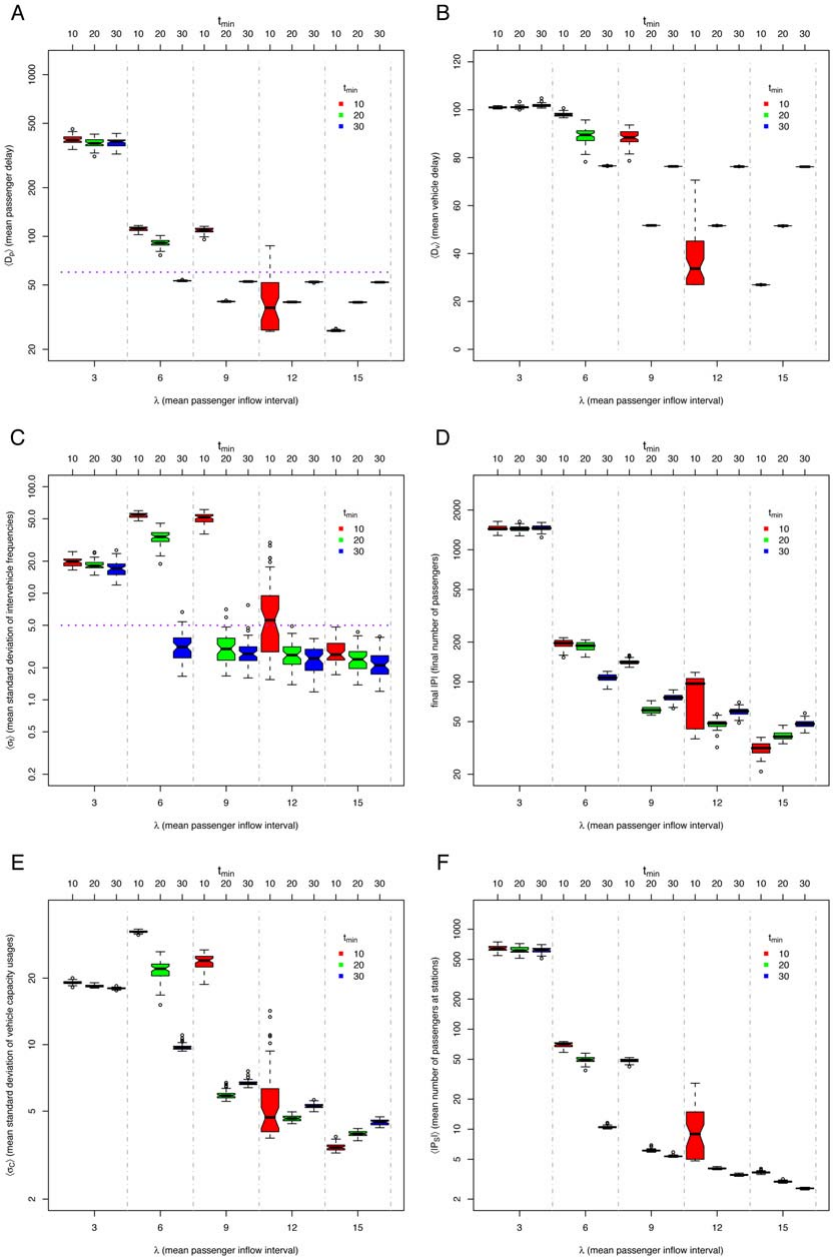
Simulation results for *minimum* method, varying mean passenger inflow intervals 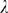 (lower 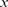 axis, separated by vertical dashed lines), different *minimum* station waiting times 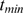 (upper 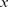 axis, also indicated by color of boxes), and number of vehicles 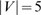: (A) mean passenger delay 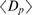 (data above horizontal dotted line indicates poor system performance), (B) mean vehicle delay 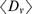, (C) mean standard deviation of intervehicle frequencies 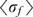 (data above horizontal dotted line implies unstable headways), (D) final number of passengers 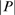, (E) mean standard deviation of vehicle capacity usage 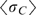, and (F) mean number of passengers at stations 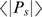.

Results of simulations for varying 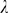 and 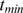 are shown in Figure 5. We can see that the *default* method, *i.e.* with no restrictions, performs poorly for all passenger densities compared to the *minimum* method. However, different values of 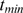 give best results at different passenger densities (See Figures 5A and 5B): For 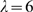 (high passenger densities) the best value is 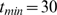, for 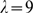 the best value is 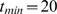, and for 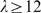 (low passenger densities) the best value is 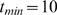. If there are few passengers, a smaller 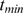 value leads to smaller passenger and vehicle delays. Nevertheless, larger values of 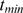 provide more stable equal headway distributions. It is precisely when the standard deviation of the intervehicle frequencies 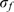 “jumps”, *i.e.* 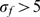 (See Figure 5C) that the delays and the vehicle capacity unbalance also jump (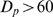 in Figure 5A and 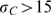 in Figure 5E). However, notice that more stable headways do not imply a better performance. *E.g.* for 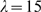, more stable headways (lower 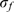) have greater passenger delays 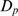. This is also the case for the unbalance of vehicle capacity usage 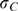 (Figure 5E), which is more correlated with 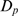 than with 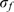. Still, we can say that the system “breaks down” when the equal headway becomes unstable. The points at which the system breaks down for different 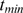 values are determined by the amount of passengers waiting at the stations: if vehicles need to spend more than 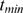 time at stations to allow the descent and boarding of passengers, some vehicles will be delayed, platooning will occur, and the performance of the system will be comparable to that of the *default* method. The cause of the equal headway instability is that vehicles are delayed by passengers and go slower than expected. At a value of 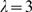, the system capacity is exceeded (see Figure 5D) and more passengers arrive at stations than those the system is able to service.

### The *maximum* method

Since the *minimum* method did not improve the system performance consistently nor maintained always equal headways for long periods of time, a further restriction was added: vehicles stay a *maximum* time 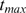 at stations, unless there are passengers still exiting the vehicle. When the waiting time at a station reaches 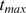, the vehicle departs, even if there are passengers waiting at the station. This attacks the problem of vehicles going slower than expected. We called this the *maximum* method. There is still a 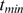 time, so vehicles will wait at stations sometime in the interval 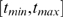 if 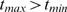 or *exactly* 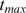 if 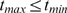, unless there are more than 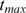 passengers exiting at a single station (remember they take one tick each). In this case, the vehicle will depart as soon as all the passengers have exited, without admitting any new passengers.

**Figure 6:**
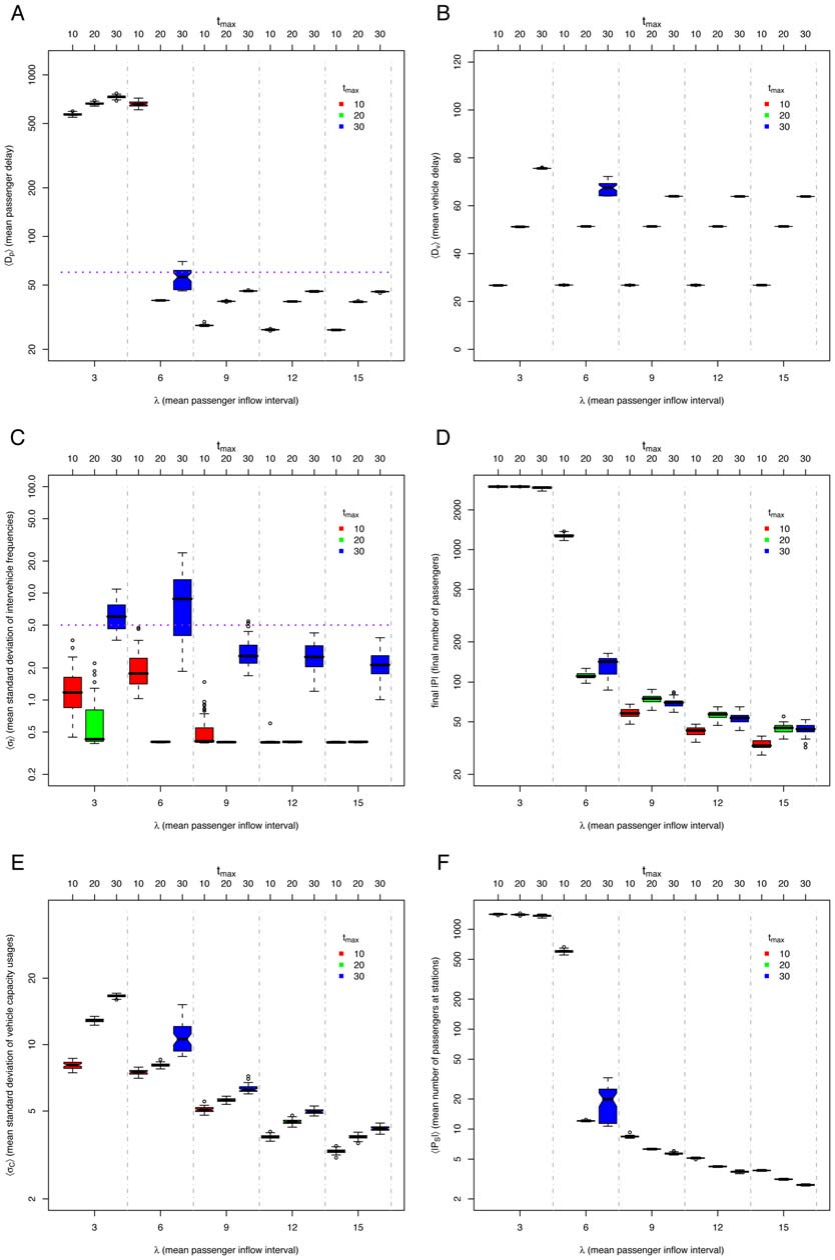
Simulation results for *maximum* method, varying mean passenger inflow intervals 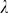 (lower 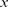 axis, separated by vertical dashed lines), different *maximum* station waiting times 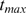 (upper 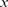 axis, also indicated by color of boxes), and number of vehicles 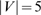: (A) mean passenger delay 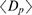 (data above horizontal dotted line indicates poor system performance), (B) mean vehicle delay 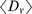, (C) mean standard deviation of intervehicle frequencies 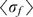 (data above horizontal dotted line implies unstable headways), (D) final number of passengers 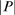, (E) mean standard deviation of vehicle capacity usage 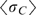, and (F) mean number of passengers at stations 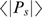.

We performed a similar set of simulations as for the previous methods. The results are shown in Figure 6. For all cases, a 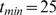 was used.

We can see from Figure 6C that when 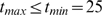, equal headway is maintained for all 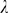 values. This leads to an even vehicle capacity usage in most cases (See Figure 6E). However, low values of 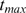 lead to an early saturation of the system (*e.g.* 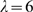, 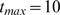 in Figures 6D and 6F), since vehicles stop for reduced times. Thus, passengers accumulate at stations. Even when the goal of equal headway is achieved, the performance is bad when 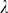 saturates the capacity of the system for a specific 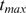 value. Still, using the best 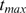 value is chosen for a particular 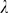, the *maximum* method improves considerably the passenger delays 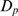 (See Figure 6A) and vehicle delays 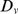 (See Figure 6B) compared with the *default* method, except when the system saturates (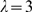). For 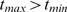, performance is similar to that of the *minimum* method. In this case, equal headways are roughly maintained for 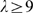. When 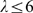, the trains are delayed by the number of boarding passengers waiting at stations, which is reflected in increased vehicle delays 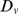 (See Figure 6B).

Another way of comparing the three methods presented so far is with the mean number of passengers at stations 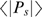 for a given 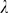 (See Figures 4F, 5F, and 6F). If 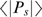 is low, it implies that the service is efficient.

We performed further simulations using heterogeneous interstation distances 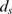 and obtained results similar as those presented above.

### Adaptive methods

A quick examination of Figures 5A and 6A shows that there are different best values of 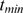 and 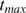 for different passenger densities, *i.e.* 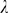, for both *minimum* and *maximum* methods. To exploit this feature, adaptive methods were developed to decide automatically which values are best to use at a specific time.

**Figure 7:**
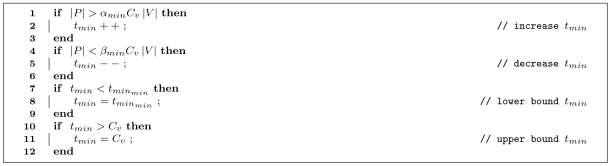
Pseudocode for the self-regulation of 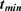. If the total number of passengers in the system 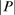 is greater than the total capacity of all vehicles (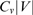) multiplied by a factor 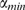, then 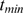 is increased (line 2). To decrease 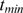 (line 5), a similar relation is taken into account, namely when the number of passengers is lesser than the system capacity multiplied by a factor 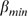. Lines 7–12 bound 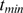, not to be smaller than 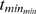 nor greater than the capacity of a single vehicle 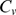.

Figure 7 shows the self-regulation mechanism for 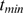. If the total number of passengers 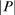 in the simulation exceeds the total capacity of the vehicles (this is the capacity of a single vehicle 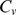 multiplied by the number of vehicles 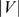), multiplied by a factor 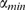, then 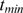 is increased (line 2). This is because for more passengers 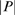 (inversely correlated with 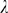), a higher value of 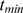 performs best. On the other hand, if 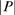 is lesser than the total capacity of the vehicles multiplied by a factor 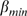, 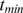 is decreased (line 5). This occurs when there are few passengers accumulating at stations, so vehicles can service them better with a lower 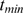. However, the 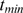 should be bounded, not to decrease too much, so it cannot be lesser than a parameter 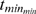 (line 8). Correspondingly, 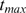 is bounded not to be greater than the time it takes an empty vehicle to fill up. In our model, this is the vehicle capacity 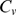, as 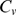 passengers will fill up an empty vehicle in 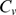 ticks. Even if there are passengers at a station, there is no point in increasing 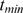, since vehicles will be full by that moment.

**Figure 8:**
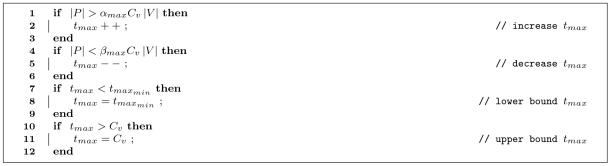
Pseudocode for the self-regulation of 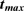. The rationale for adjusting 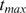 is very similar to that of Figure 7: it is increased when there are many passengers in the system, it is decreased when there are few, and it is bounded.

The self-regulation for 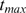 is done in a very similar way, described by Figure 8.

After a careful parameter exploration, the values chosen for the simulations were 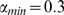, 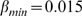, 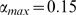, 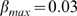, 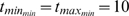 ticks, and 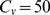 passengers. These parameters achieved a proper balance between the waiting times and the passenger demand: if there are few passengers, the waiting times should be low, whereas waiting times should be extended for an increased passenger demand. The speed in which these parameters are adjusted is also important: on the one hand, if it is too slow, then there will be a waste of resources. On the other hand, if the speed is too fast, the waiting times will be adjusted before the system reaches a balance, so it might overshoot the adjustments. The 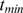 or 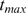 values were updated with the algorithms shown in Figures 7 or 8 every 100 ticks.

**Figure 9:**
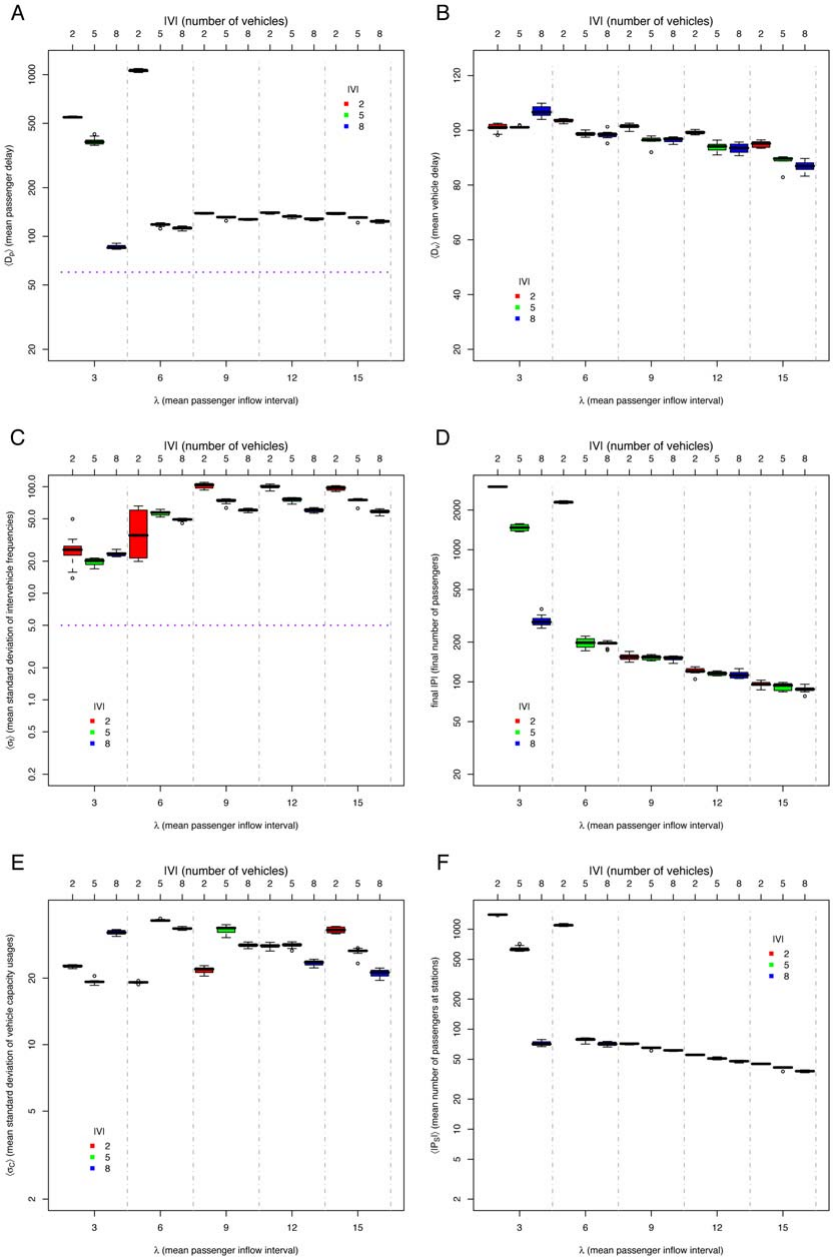
Simulation results for *default* method, varying mean passenger inflow intervals 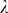 (lower 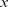 axis, separated by vertical dashed lines) and number of vehicles 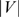 (upper 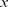 axis, also indicated by color of boxes): (A) mean passenger delay 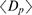 (data above horizontal dotted line indicates poor system performance), (B) mean vehicle delay 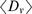, (C) mean standard deviation of intervehicle frequencies 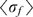 (data above horizontal dotted line implies unstable headways), (D) final number of passengers 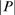, (E) mean standard deviation of vehicle capacity usage 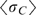, and (F) mean number of passengers at stations 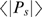.

**Figure 10:**
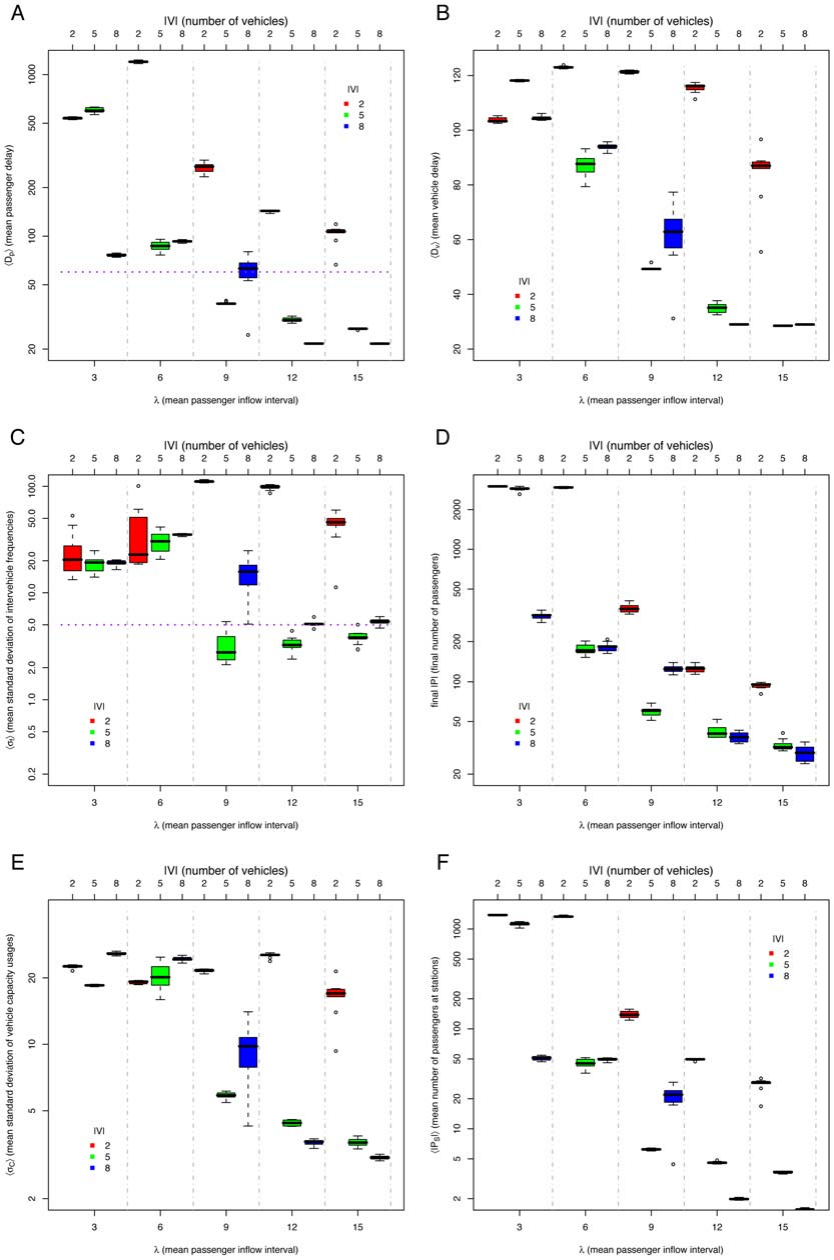
Simulation results for *adaptive minimum* method, varying mean passenger inflow intervals 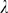 (lower 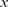 axis, separated by vertical dashed lines) and number of vehicles 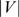 (upper 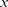 axis, also indicated by color of boxes): (A) mean passenger delay 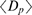 (data above horizontal dotted line indicates poor system performance), (B) mean vehicle delay 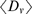, (C) mean standard deviation of intervehicle frequencies 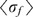 (data above horizontal dotted line implies unstable headways), (D) final number of passengers 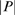, (E) mean standard deviation of vehicle capacity usage 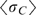, and (F) mean number of passengers at stations 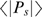.

**Figure 11:**
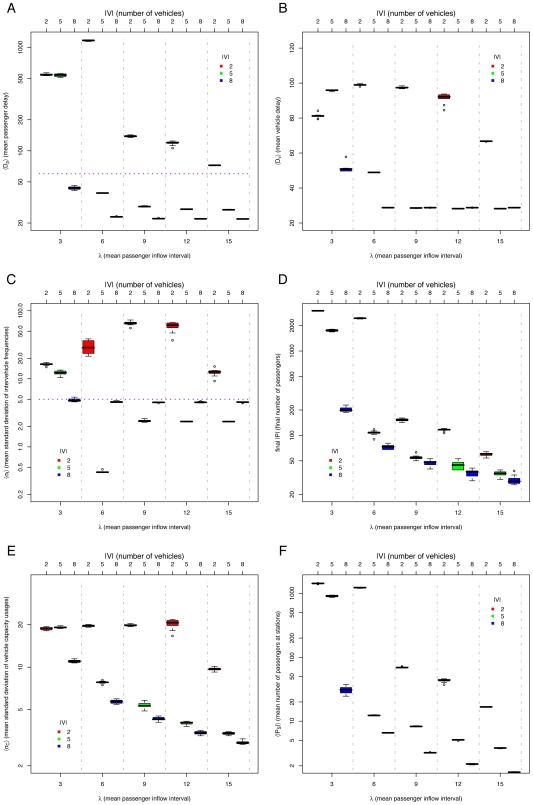
Simulation results for *adaptive maximum* method, varying mean passenger inflow intervals 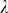 (lower 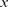 axis, separated by vertical dashed lines) and number of vehicles 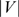 (upper 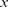 axis, also indicated by color of boxes): (A) mean passenger delay 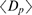 (data above horizontal dotted line indicates poor system performance), (B) mean vehicle delay 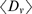, (C) mean standard deviation of intervehicle frequencies 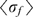 (data above horizontal dotted line implies unstable headways), (D) final number of passengers 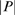, (E) mean standard deviation of vehicle capacity usage 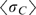, and (F) mean number of passengers at stations 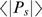.

Figures 9, 10, and 11 show results of simulations comparing the *default*, *adaptive minimum*, and *adaptive maximum* methods, for different number of vehicles 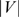. The *adaptive maximum* method keeps a fixed 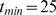 ticks. For these sets of simulations, the boxplots show statistics of ten runs per box.

We can see from Figures 9D, 10D, and 11D that the system saturates roughly independently of the method used for 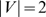, 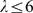 and 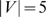, 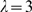. For 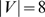 the system does not saturate for the values of 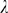 explored.

The *adaptive maximum* method manages to maintain the most stable headways (See Figures 9C, 10C, and 11C). Also, the best performance is achieved by the *adaptive maximum* method, which is reflected in the lowest passenger delays 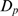 (See Figures 9A, 10A, and 11A), vehicle delays 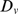 (See Figures 9B, 10B, and 11B), uneven vehicle capacity usage (See Figures 9E, 10E, and 11E), and lower accumulation of passengers at stations (See Figures 9F, 10F, and 11F). Note that the *adaptive maximum* method always achieves an equal headway, unless the passenger density is high enough to force vehicles to wait at stations more than 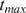 due to exiting passengers, as shown in Figure 11C. The *adaptive maximum* method overcomes the problem of the *maximum* method where the system was saturated when vehicles were departing stations too soon and were not serving the passenger demand.

**Figure 12:**
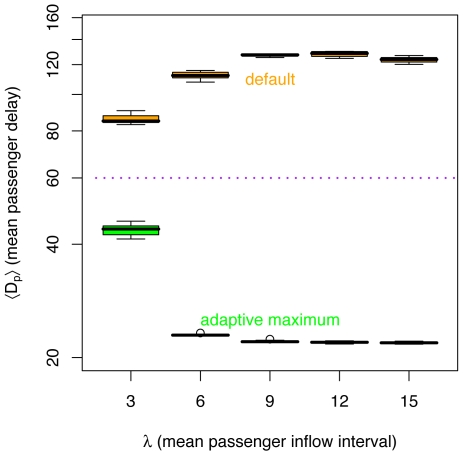
Comparison of passenger delays 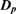 between *default* and *adaptive maximum* methods, varying mean passenger inflow intervals 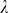 and 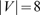. For 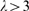, the mean delays for the *default* method are about six times larger than those for the *adaptive maximum* method (notice log scale on 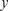 axis).

Since the *default* method offers similar delays independently of the passenger density, adding more vehicles at peak hours has negligible effects (see in Figure 9A that passenger delays 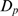 are similar, independently of the number of vehicles 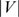), unless the system saturates. However, since improvements over the *default* method are greater for lower passenger saturation rates, adding vehicles at peak hours offers much greater benefits with the *adaptive maximum* method. With more vehicles available, the *adaptive maximum* method is able to consistently reduce passenger delays. Depending on the number of vehicles, the improvement can be greater. For example, for eight vehicles, the passenger delays can be reduced to about one sixth of the delay obtained with the *default* method for 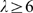 (see Figure 12).

**Figure 13:**
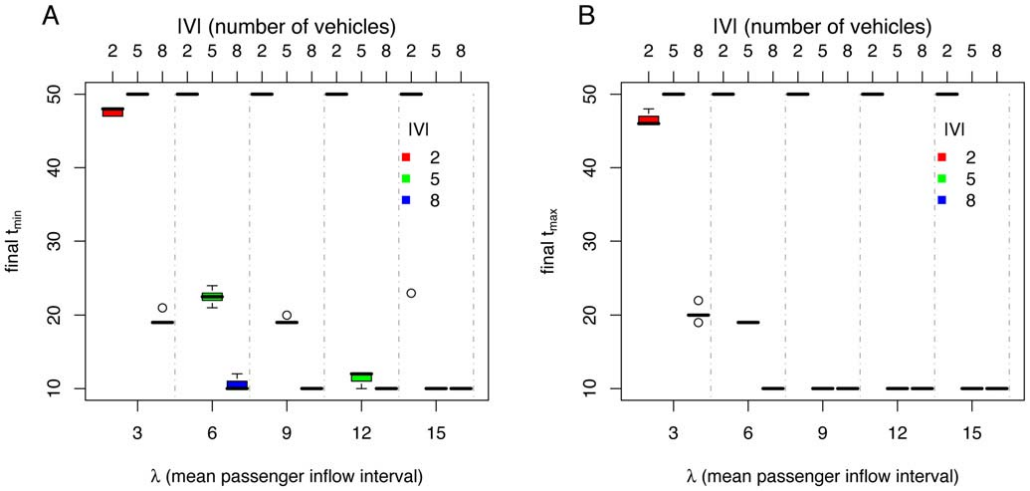
Simulation results for *adaptive minimum* and *adaptive maximum* methods, varying mean passenger inflow intervals 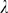 (lower 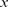 axis, separated by vertical dashed lines) and number of vehicles 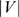 (upper 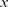 axis, also indicated by color of boxes): (A) Final 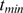, (B) final 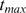 (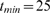).

To observe how the *adaptive* versions of the *minimum* and *maximum* methods regulate 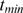 and 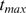 respectively, Figure 13 shows their final values in the simulations discussed above. We can see that for low passenger demands (*i.e.* high values of 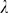), the methods self-regulate to low values and vice versa. Note that this seems not to be the case for 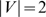 and 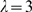. This is because the system saturates quickly and the simulation stops before the parameters have reached their maximum value.

Notice also that the final values for 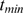 and 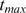 are very similar. This suggests that the adaptive methods are able to find by themselves values close to the optimal waiting times at stations for a given 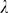. In other words, for a given passenger density, the system will maintain headways and be most efficient if each vehicle spends a particular time at each station. On the one hand, vehicles will not go faster or slower than average, maintaining equal headways. On the other hand, vehicles will spend enough time at stations to serve the current passenger demand: not more (wasting time idling at stations) and not less (vehicles departing stations while passengers accumulate). Thus, theoretically there are optimal waiting times for different densities, and the methods presented above are able to find values close to them, even as the passenger demand changes. In practice, however, there might be other things to be considered.

## Discussion

The proposed model has similar aspects to Nagatani's cyclic tram model [23] and O'Loan et al. 's bus route model [24]. They observed a phase transition between a homogeneous phase, *i.e.* equal headways and an inhomogeneous “jammed” phase, *i.e.* unstable equal headways. This transition depends on the availability of noise, which in our model is delivered by random arrival of passengers. This is consistent with our results, where the *default* method is always in the “jammed” phase (See Fig. 4C). The above cited models and others, *e.g.* [25]–[28], have focussed on the dynamic aspects of bus or tram systems, not so much on exploring potential solutions to prevent equal headway instability. An exception, Nagatani [29] studied the option of full buses not stopping at certain stops, which helps reduce the equal headway instability. However, this is problematic for passengers exiting at those stops.

Since the *default* method always leads to an equal headway instability, restrictions are required to prevent it. The *minimum* method attacks the problem of vehicles going faster than expected and works for low 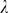 values relative to 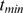. Additionally, the *maximum* method attacks the problem of vehicles going slower than expected and achieves a better headway stability by forcing vehicles to spend specific times at stations. However, this might be difficult to implement (see below). Even when headway stability can be maintained, a low 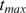 can lead to an early system saturation. Thus, headway stability by itself does not imply a good system performance. This is seen again in the case described in the next paragraph.

One alternative to promote equal headways could be to keep a longer minimum intervehicle distance 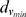. The idea behind this is to force equal headways via equal distances. This works if stations are spaced homogeneously and 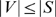: if fast vehicles are forced to wait behind fast ones (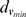 slightly smaller than 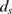), then they will not be able to aggregate and the stations loads will be balanced, giving a similar performance to the *adaptive maximum* method. However, if 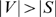 or stations are not homogeneously spaced, vehicles are forced to wait between stations, leading to greater delays than those of the *default* method with 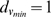. If 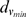 has a medium value, *i.e.* 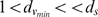 then vehicles still aggregate, although not as close as with 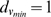. Note that if 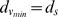, then all vehicles go at the speed of the slowest one, *i.e.* the delay of a single vehicle affects instantly the whole system.

Vehicles serving at full capacity deliver greater delays, *i.e.* heterogeneous vehicle usage lead to heterogeneous headways. For this reason, one ingredient to promote equal headways is to have space available in arriving vehicles, *i.e.* similar loads will lead to similar travel times for vehicles along a route, which will lead to similar headways. An efficient method, such as the *adaptive maximum* method, can use the availability of extra vehicles to reduce passenger delays by balancing loads. A method without restrictions, such as the *default* method, cannot exploit extra vehicles, as these travel mainly empty, following closely slower vehicles servicing at full capacity.

We have studied methods to maintain an equal headway distribution in an abstract scenario. How to implement *e.g.* maximum waiting times at stations is a tough practical question, since passengers may be eager to board a vehicle and not allow its departure on time, risking their own safety. One could think of physical barriers to regulate and mediate the boarding of passengers. These might be costly and would need to be specifically engineered for the passenger behaviors of different cultures. There can be several mechanisms that promote equal headways, not necessarily the ones explored here. We were not interested in finding a practical method, but in studying the effect of equal headways in public transport system performance. Moreover, such methods need to consider the peculiarities of a specific implementation, absent in our model. Here we limit ourselves to suggest recommendations that contribute to maintain an equal headway.

### Recommendations

Based on the explorations of the equal headway instability phenomenon with our model, we can suggest the following recommendations.

For passengers:

- If a crowded vehicle arrives at a station after a long waiting time, it is very probable that empty vehicles are coming close behind. Do not board the crowded vehicle, contributing to its further delay and of all the passengers within. If even some people follow this advice, it is likely that crowded vehicles will be able to go relatively faster, allowing the vehicles behind them also to go faster, improving the performance of the whole system. Waiting at the station for another vehicle might actually contribute to a faster trip.
- Give way to people descending a vehicle before boarding. Trying to “win” and enter before others will delay everybody. Sometimes waiting for a second or a third vehicle is faster than attempting to board a crowded one (especially in transport systems that allow passing).
- Inside a crowded vehicle, go far from the doors. Giving space to ascending and descending people will accelerate the travel. Make way to the doors not too long before exiting.

For engineers:

- It makes little sense to add vehicles if these are not regulated to maintain an equal headway.
- Design methods to regulate equal headways. This will improve considerably the system performance. The most common method is to have scheduled arrival and waiting times at stations, with margins for adjustment along the route and also at terminals.
- Educate passengers with publicity campaigns to promote equal headways. In many cases these cannot be achieved because of passenger behavior. Explain to passengers the equal headway instability phenomenon, indicating that following certain norms will help them arrive earlier and more comfortably at their destination. Suggest recommendations as those outlined above, adapted to the local culture.

### Other transport systems

Equal headway instability can also be triggered by traffic or traffic lights. Here we studied the simplest case, where delays are caused only by passengers boarding at stations. This would apply to metros, some trams, trains, and elevators. Buses and some trams interact with traffic and this can affect considerably their performance. Bus rapid transit systems and some trams have dedicated lanes or tracks, so in principle they are not affected by the traffic density. However, traffic lights can trigger an equal headway instability. A tentative option would be to use self-organizing traffic lights [30] to give priority to public transport vehicles without affecting the flow of other vehicles in a city.

The recommendations posed above apply also to other types of transport system. They will not prevent by themselves equal headway instabilities, but different ingredients contribute to improving the performance of systems by making equal headways more stable.

### Conclusions

Equal headway instability is a general phenomenon, independent of peculiarities of the transport route and type. We presented a model that is able to reproduce qualitatively the properties of the equal headway instability. The two methods and their adaptive versions proposed restrictions that promote equal headways, reducing considerably delays and improving system performance.

This problem has many technological solutions. However, after a careful examination, we can see that there is an additional social problem: passenger behaviors. Recommendations were made for passengers and engineers to promote equal headways. Technology is not sufficient to achieve this. Social campaigns are required, since passenger behavior affects considerably the time vehicles spend at stations, in some systems their only source of delay.

As a future work, we intend to apply our results to different public transportation systems in Mexico City: the Metro and the bus rapid transit systems Metrobus and Pumabus (operating within the UNAM's main campus), all suffering from equal headway instabilities. This will require a refinement of the model for the specific domains, to compare simulation results with real data, to study passenger behaviors, and to collaborate with the authorities overseeing and regulating these systems.
